# In vitro and in vivo assessment of the antioxidant potential of isoxazole derivatives

**DOI:** 10.1038/s41598-022-23050-x

**Published:** 2022-10-29

**Authors:** Mohammed Hawash, Nidal Jaradat, Murad Abualhasan, Manar Thaher, Rawan Sawalhi, Nadeen Younes, Amani Shanaa, Mariam Nuseirat, Ahmed Mousa

**Affiliations:** 1 Department of Pharmacy, Faculty of Medicine and Health Sciences, An-Najah National University, Nablus, Palestine; 2 Department of Biomedical Sciences, Faculty of Medicine and Health Sciences, An-Najah National University, Nablus, Palestine

**Keywords:** Drug discovery, Structural biology, Medical research, Molecular medicine, Chemistry

## Abstract

Previously developed fluorophenyl-isoxazole-carboxamides derivatives were re-synthesized and their scavenging activity against DPPH free radical and inhibitory activity against lipase and α-amylase enzymes were evaluated. The inhibition of the tested enzymes was weak while the most potent activities were observed in the DPPH assay. In particular, compounds **2a** and **2c** demonstrated high antioxidant potency with IC_50_ values of 0.45 ± 0.21 and 0.47 ± 0.33 µg/ml, respectively, when compared to Trolox, the positive control compound, which has an IC_50_ value of 3.10 ± 0.92 µg/ml. Based on the in vitro results, the most potent compound **2a** was chosen for in vivo evaluation of antioxidant properties using 20 male mice injected intra-peritoneally and divided into four groups. The in vivo results revealed that total antioxidant capacity (TAC) obtained for mice treated with **2a** was two folds greater than that of mice treated with the positive control Quercetin. Although further biological and preclinical investigations need to be performed to assess the therapeutic potential of **2a**, the results of this study show promising antioxidant activities both in vitro and in vivo.

## Introduction

Many dangerous pathophysiological processes, such as diabetes, cancer, neurological disorders, cardiovascular disease, and obesity, are exacerbated by oxidative stress^1^. In addition, the increased free radical production can result in an imbalance of oxidant and antioxidant activity, which is at the heart of oxidative stress^2–4^. A free radical is an atom or molecule with one or more unpaired electrons produced by a living organism in response to environmental stress to maintain cellular hemostasis^2,3,5^. Usually, these free radicals are produced at a very low level, but when pathological conditions or environmental stress occur, production quickly increases and becomes highly destructive to cells^3^.

**Figure 1:**
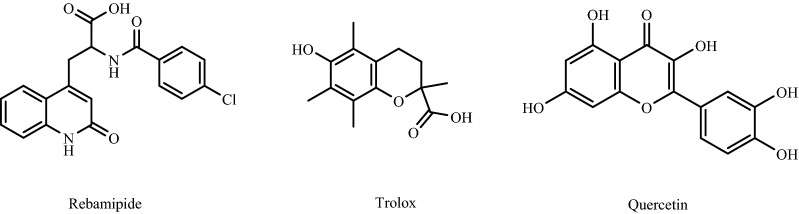
Chemical structures of antioxidant agents (Rebamipide, Trolox and Quercetin).

Antioxidant defense mechanisms are present in all biological systems to eliminate the harmful effects of oxidative stress. Antioxidants are substances that supply electrons to damaged cells to prevent and stabilize free radical damage. They also convert free radicals into waste by-products that the body eliminates^6^. There are two types of antioxidants; enzymatic (endogenous) and non-enzymatic (exogenous; mainly from food). Catalase (CAT) and glutathione peroxidases (GPX) are two of the most significant enzymatic antioxidants. Micronutrients (metals and vitamins) are the most common non-enzymatic antioxidants. For example, vitamin C (ascorbic acid) is a water-soluble vitamin; vitamin E (tocopherol) is a lipid-soluble vitamin. Copper and iron metals and ferritin that contains iron metal; all of them act as coenzymes^7^. Several additional substances, such as albumin, have been shown to have extraordinary free radical scavenging activities, including Quercetin^8^, Rebamipide, and Trolox (Fig. 1)^9^, which are considered potent antioxidant compounds. Other many new compounds with significant antioxidant activity have been synthesized, such as quinolinone-3-aminoamide, thienopyrimidine, thienopyrazole, and *N*-aryl-1,4-dihydropyridine derivatives^10^.

Isoxazole derivatives are chemicals with significant pharmacological properties. Many drugs with an isoxazole structure have been shown to have a wide range of biological effects, such as antituberculosis, analgesic, antipyretic, anti-inflammatory, antiplatelet, anti-HIV, antifungal, antibacterial, antioxidant, and anticancer effects^11,12^. The evaluated isoxazole compounds in this study (**2a-2e**) were synthesized by our research team as anticancer agents, and compound **2e** was the most potent compound against Hep3B and HepG2 cancer cell lines with IC_50_ values of 5.76 and 34.64 µg/ml, respectively as well as compounds **2a, 2b** and **2d** showed potent inhibitory activity against Hep3B with an IC_50_ values 9.58, 8.54 and 7.66 µg/mL. Moreover, **2b** and **2e** compounds reduced the necrosis rate of Hep3B to 4-folds and shifted the cells to apoptosis^13^. Thus, our previous study suggested that **2a-2e** might be potential and promising anticancer agents against hepatocellular carcinoma prompting us to investigate their mechanism of action as well as their anti-obesity and antidiabetic potential. Accordingly, in the current study the fluorophenyl-isoxazole-carboxamide derivatives **2a-2e** were re-synthesized and their antioxidant activity (in vitro and in vivo) was evaluated together with their inhibitory activity against lipase and α-amylase enzymes.

## Results and discussion

### Chemistry

**Figure 2:**
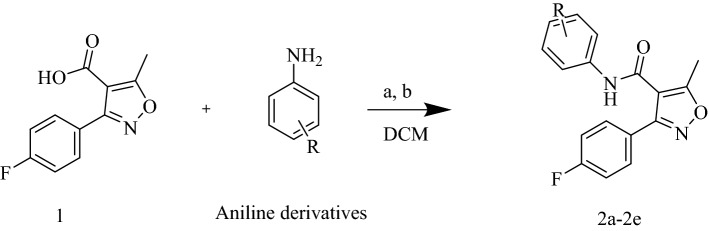
1 + aniline derivatives stirred in 17 ml Dichloromethane (DCM) as solvent, **a** DMAP and **b** EDC all under inert gas and stirred for 72 h.

The synthesis of fluorophenyl-isoxazole-carboxamide derivatives (**2a-2e**) was outlined in Fig. 2. EDCI and DMAP were used as coupling and activator reagents^14^. The chemical structures of these synthesized derivatives were confirmed by HRMS and ^1^H-NMR, and all evaluated compounds matched the results of our last publication of these compounds^13^.

### Biological evaluations

#### In vitro antioxidant antidiabetic and anti-obesity evaluation of 2a-2e compounds

To determine the antioxidant activities, the 2,2-Diphenyl-1-picrylhydrazyl (DPPH) was used, which is considered one of the most widely used methods for measuring the in vitro antioxidant activity of various chemical compounds. This colorimetric method is accurate, easy to perform, and economical, providing a screening of the general activity of the antioxidants and is based on a stable and synthetic radical, DPPH^15^.

When DPPH reacts with an antioxidant compound, its free radical property is lost and its color changes from violet to yellow. The decrease in absorbance at 517 nm induced by antioxidants determines the reduction ability of DPPH. All the compounds showed moderate to potent free radical scavenging activity near or better than Trolox (positive control) Table 1. Compound** 2a** and** 2c** showed potent antioxidant activity with an IC_50_ value of 0.45 ± 0.21 and 0.47 ± 0.33 µg/ml, respectively, compared with the positive control Trolox (IC_50_ = 3.10 ± 0.92 µg/ml).

**Table 1:** IC_50_ values (µg/ml) of Fluorophenyl-isoxazole-carboxamide compounds and positive controls on DPPH, α-amylase, and lipase enzyme.

		IC_50_ (µg/ml)		
Code	R Group	DPPH	Lipase	α-Amylase
**2a**		0.45 ± 0.21	> 400	> 500
**2b**		43.85 ± 0.71	358.18 ± 1.32	> 500
**2c**		0.47 ± 0.33	> 400	> 500
**2d**		5.11 ± 0.91	303.90 ± 2.2	> 500
**2e**		33.39 ± 0.57	> 400	> 500
**+ ve control**		3.10 ± 0.92^a^	12.30 ± 0.33^b^	28.84 ± 1.22^c^

*P* value ≤ 0.05.

^a^Trolox,

^b^Orlistat,

^c^Acarbose.

**Figure 3:**
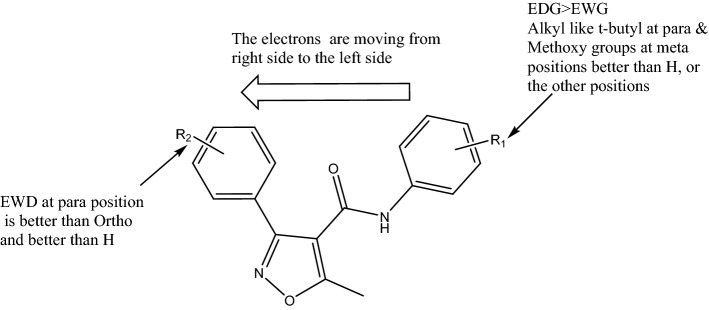
Structure–activity relationship of isoxazole derivatives.

Radical scavenging activity is one of the most well-known mechanisms of action of antioxidant compounds, and usually the reactive free radical abstracts the H atom from the antioxidant^16^. The chemical difference between the evaluated compounds was in the substituted groups on phenyl ring like electron donating (alkyl and methoxy) and withdrawing groups (halogen; Cl), the most potent compound with the lowest IC_50_ values was** 2a** compound, which contains *t*-butyl group. However, moving the electrons from the right side of our compounds (Fig. 3) to the left side can help the free radical abstract the H atom from donating groups like *t*-butyl or methoxy groups. We previously reported that the most active compound has *t*-butyl in similar series of isoxazole (*N*-(4-(*tert*-butyl)phenyl)-3-(2-chlorophenyl)-5-methylisoxazole-4-carboxamide with IC_50_ value of 7.8 µg/ml^17^, the activities were lower than the current compound (**2a**), because of the halogen at R2 (Fig. 3) was at ortho position and now is at para position, as well as in another work done by our team, we tried to evaluate similar series of isoxazole without halogen at R2 against DPPH and this series showed very weak or negligible activities as antioxidants^18^. From this data analysis of structure–activity relationship, we can conclude that the presence of electron donating group (EDG) at R1 is better than electron-withdrawing group (EWG) Fig. 3. Having analkyl group like *t*-butyl at para or methoxy at meta positions are the best for activity. A presence of EWG at R2 is better than H as well as para position is better than the ortho position.

**Figure 4:**
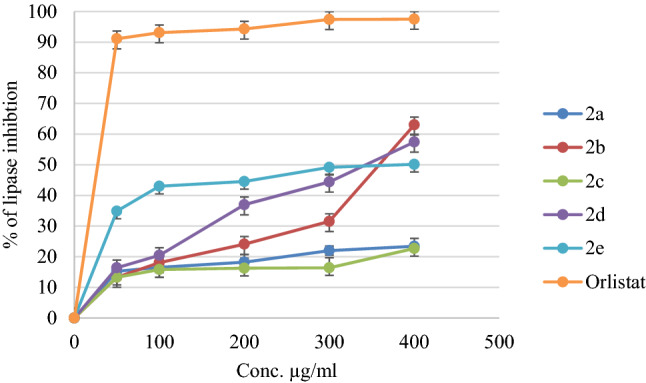
The Inhibition % of lipase that induced by compounds (**2a-2e**) and the positive control (Orlistat).

The anti-obesity activities were evaluated by measuring lipase inhibition percentages of the compounds, and the obtained results were compared with positive control Orlistat, a pharmaceutical anti-obesity drug. The results of inhibitory percentages for the tested compounds and Orlistat are shown in Fig. 4, and the IC_50_ values were calculated and listed in Table 1. All compounds showed weak or negligible activities, and the most active compounds against lipase enzyme were compounds** 2d** and** 2b** with IC_50_ values of 303.90 ± 2.2 and 358.18 ± 1.32 µg/ml, respectively, compared with orlistat IC_50_ value of 12.30 ± 0.33 µg/ml.

**Figure 5:**
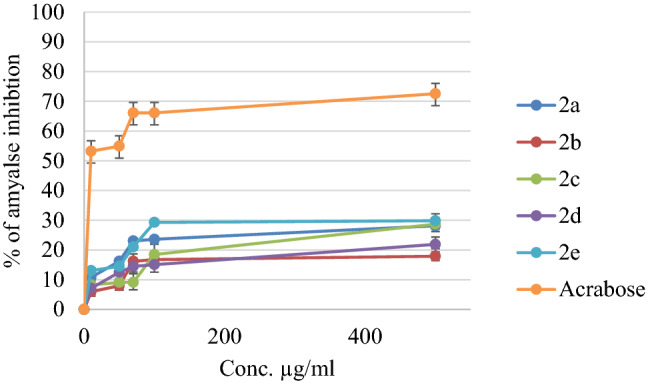
The Inhibition % of synthesized compounds (**2a-2e**) and Acarbose (positive control).

Hyperglycemia is usually controlled by different treatment protocols, one of these protocols is the inhibition of the α-amylase enzyme^19^. This enzyme is responsible for the hydrolysis of starch into simple monosaccharides^20–22^. The antidiabetic potential of the evaluated compounds was investigated by assessing their α-amylase inhibitory effects, and Acarbose, an antidiabetic drug, was used as a positive control. An evaluation of the α-amylase inhibitory activity of each compound is shown in Fig. 5. All of the evaluated compounds’ IC_50_ values were higher than 500 µg/ml (Table 1). At the concentration of 500 µg/ml, the percentage of inhibition against this enzyme of all compounds was in the range 17.89–29.83%, compared with acarbose positive control showed 72.54% at the same concentration.

### Effects of 2a compound on TAC status (in vivo)

According to the results obtained from the in vitro antioxidant activities of the evaluated compounds, the most potent compound** 2a** (*N*-(4-(*tert*-butyl)phenyl)-3-(4-fluorophenyl)-5-methylisoxazole-4-carboxamide) was selected for the in vivo antioxidant evaluation to determine the effect of it on the cumulative effect of all antioxidants present in the blood of mice. For this purpose, the TAC was determined using a commercially available assay kit (Abcam ab65329), in four groups of mice: **2a** treated (5 mg/kg over 6 days, n = 5), **2a** treated (10 mg/kg over 6 days, n = 5), quercetin treated (positive control; 10 mg/kg over 6 days, n = 5), and vehicle-treated (negative control; n = 5). All mice were sacrificed seven days after the treatment (or the first administration of the vehicle).

**Figure 6:**
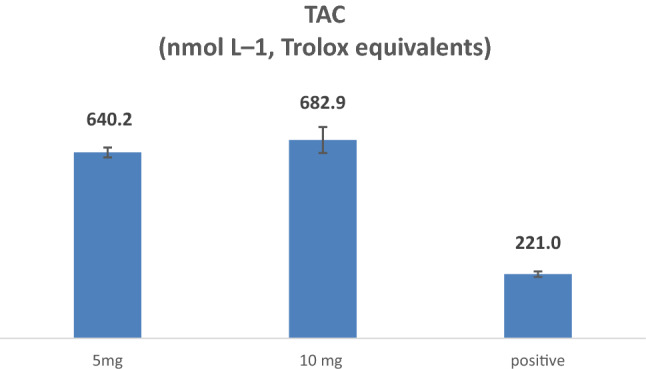
the TAC levels of (**2a**) compound at 5 and 10 mg/ kg doses in comparison with positive control quercetin (10 mg/kg).

The absorbance values of the used kit were plotted against a serial concentration of Trolox standards in the concentration range of (4–20 nM). The calibration curve regression line equation was found to be **Y = 0.0533x—0.1293**. The calibration curve showed linearity with R^2^ = 0.9688. The generated regression line equation was utilized to calculate the Trolox equivalent total antioxidant activity of the injected compound and positive quercetin control of the experimental mice. The average results of the total antioxidant activity of ten replicates of plasma samples were reported. The results showed that in mice treated with** 2a** compound at both doses (5 and 10 mg/kg), their TAC levels were increased, compared to the vehicle, and positive control quercetin in the plasma. The ANOVA statistical test of the antioxidant result showed a significant difference with a *P* value < 0.05. The summary of the results is shown in (Fig. 6).

The in vivo result reflects the in vitro antioxidant activity of the evaluated compound *N*-(4-(*tert*-butyl)phenyl)-3-(4-fluorophenyl)-5-methylisoxazole-4-carboxamide (**2a**). In a similar study by Nuntiya Somparn et al., they studied the in vivo antioxidant activity of aqueous lemongrass extract (CCW) injected at a concentration of 1000 mg/kg having a TAC of 1.82 mM ^23^.

The results obtained with our tested compound showed much more potent antioxidant activity at lower concentrations compared to the positive control, that demonstrate a promising antioxidant activity, which requires further studies, including the synthesis of a series of analogs of the active compound **2a**.

## Conclusion

In this study, we tested the in vitro and in vivo antioxidant activity of the isoxazole-carboxamide derivatives** 2a-2e** , previously reported as potential anticancer agents, togheter with their ability to suppress the effects of lipase and amylase enzymes.The tested compounds resulted weak enzymatic inhibitors while showed potent scavenging activity against DPPH free radical. Compound** 2a** resulted the most active compound both in vitro and in vivo against free radical formation. Despite **2a** is characterized by low water solubility, it can be dissolved with a non-toxic concentration of propylene glycol and resulted more potent than Quercetin, the reference compound, in the in vivo assays. The results obtained pave the way for the future investigation/optimization of the therapeutic potential of the new indentified lead** 2a**.

## Experimental section

### Chemicals

All used chemical and biological reagents were purchased from Alfa Aesar, Sigma-Aldrich, and Frutarom (UK): 3-(4-fluorophenyl)-5-methylisoxazole-4-carboxylic acid, dichloromethane (DCM), dimethyl aminopyridine (DMAP), 1-Ethyl-3-(3-dimethylaminopropyl) carbodiimide (EDCI), DPPH, Na_2_HPO_4_\NaH_2_PO_4_, Trolox, methanol, starch, porcine pancreatic amylase, lipase, dinitrosalicylic acid (DNSA), Na_2_CO_3_, dimethyl sulfoxide (DMSO), acarbose, Abcam's total antioxidant capacity assay kit (ab65329), Quercetin, normal saline, distilled water (ddH2O), PBS, DMSO (anhydrous), and propylene glycol.

### The general synthesis procedure of Floro-isoxazole-carboxamide derivatives (2a-2e)

The 3-(4-fluorophenyl)-5-methylisoxazole-4-carboxylic acid (1.5 mmol) **(1)** was dissolved in 17 ml of DCM, and the activator reagents (DMAP 0.3 mmol, EDCI 1.8 mmol) were added and allowed to stir under inert gas at room temperature (25 °C) approximately 30 min. After that, each aniline derivative (1.8 mmol) was added individually and the mixture was allowed to stir for 72 h. Re-crystallization and flash chromatography was used to purify the final products, and all compounds were characterized by using HRMS and proton NMR to confirm their structures. The synthesis of these compounds is presented^13,24^ in Fig. 2.

### Antioxidant activity method (in vitro)

The free DPPH radical scavenging assay was used to measure the antioxidant activity of the isoxazole derivatives. A 1000 μg/ml stock solution of each compound was prepared in methanol. In addition, a 1000 μg/ml solution of Trolox was also prepared (the reference standard). A dilution series was prepared from the stock solutions for each compound, giving seven serial dilutions at 1, 2, 7, 20, 50, 80, and 100 μg/ml. One ml of each compound dilution was mixed with 1 ml 0.002 g/ml DPPH in methanol. One ml of methanol was added to give a final working volume of 3 ml. The DPPH solution was freshly prepared, as it was very sensitive to light. The blank control of the series concentrations was DPPH in methanol in a ratio of 1:2, without the addition of any compound. All working solutions were incubated at room temperature (25 °C) in the dark for about 30 min. Optical densities were then measured with a spectrophotometer at a wavelength of 517 nm. The following equation was used to calculate % DPPH inhibition for each compound, with Trolox as the standard compound:

$$ {\text{DPPH}}\;{\text{inhibition}}\;\% = \left( {{\text{A}}_{{\text{B}}} - {\text{A}}_{{{\text{ts}}}} } \right)/{\text{A}}_{{\text{B}}} \times 100\% $$

where, A_B_ is the recorded absorbance of the blank solution, and A_ts_ is the recorded absorbance of the tested sample solution^25^.

### Antioxidant activity (in vivo)

#### Animal

Mice experiments were performed in accordance with the Association for Assessment and Accreditation of Laboratory Animal Care International and approved by the Institutional Review Board of Animal House and Use Committee of the An-Najah National University (Approve number: pharm. May 2012/15). In this study, twenty healthy adult male mice (weight range: 24–33 g) were used in the experiment. They kept four per cage in the animal house. Mice were allowed to acclimatize to the animal facility for 7 days before testing under controlled conditions of temperature (22 ± 2 °C). Mice are re-used with a minimum 7 days interval between drug testing. All experiments were performed during the light portion of the day cycle. All animals fasted over the night of the experiment.

Twenty mice were divided into four groups randomly, with five mice in each. The first group was administered with our novel compound **2a** (5 mg/kg), the second group with the same compound (10 mg/kg), the third group with Quercetin (10 mg/kg) as a positive control, and the fourth group with normal saline (20%) and propylene glycol 80% as a negative control group ^26^.

### Drug administration

To test the TAC of the novel compound, four groups of mice were injected intraperitoneal (i.p.) and employed with vehicle (80% polyethylene glycol (PG) and 20% normal saline (NS) for 6 days. The first and second groups were injected with 5 and 10 mg/kg/day of **2a** compound dissolved in the same vehicle, respectively. The third group was injected with a known antioxidant agent positive control (Quercetin), with 10 mg/kg/day, and the last group was the negative control group (80% polyethylene glycol (PG) and 20% Normal saline (NS). All mice were anesthetized with ketamine (60 mg/kg) and xylazine (5 mg/kg) and sacrificed 24 h after the final injection. Then, the mouse blood sample was collected and centrifuged to collect serum. For long-term stability, serum was frozen at − 20 °C^27,28^.

### Serum collection

All mice were sacrificed after seven days of treatment by placing each mouse in a plexiglass chamber with 5% of isoflurane anesthetic drug, for 5 min, and then when the mouse was fully sedated, they were sacrificed by rapid decapitation. Trunk blood was collected in EDTA-coated tubes for use in the kit assay^29,30^.

### Sample preparation

Using DPPH assay that basis on measures the total antioxidant capacity (TAC) of compounds that can transfer hydrogen atoms. The compound (DPPH• +) is a colored and stable radical cation of purple color which shows maximum absorbance at 517 nm. Antioxidant compounds, which can transfer an electron to DMPD• + , cause a discoloration of the solution. This reaction is rapid and proportional to the antioxidant capacity of the sample. Regarding the ab65329 Total Antioxidant Capacity Assay Kit (Colorimetric), the TAC assay protocol was conducted, the Cu^2+^ ion is converted to Cu + by both small molecule and protein antioxidants. The Protein Mask prevents Cu^2+^ reduction by proteins, enabling the analysis of only the small molecule antioxidants. The reduced Cu + ion is chelated with a colorimetric probe giving a broad absorbance peak around 570 nm, proportional to the total antioxidant capacity. Assorted glassware for the preparation of reagents and buffer solutions, Tubes for the preparation of reagents and buffer solutions and 96 well plates with clear flat bottom Prepare Trolox standard by adding 20 µL of DMSO and 980 µL of H_2_O (1 ml) after that were stored in the refrigerator (− 20 °C). Six standards were prepared as following: 0 µL of Trolox + 300 µL H_2_O, 12 µL Trolox + 288 µL H_2_O, 24 µL Trolox + 276 µL H_2_O, 36 µL Trolox + 264 µL H_2_O, 46 µL Trolox + 252 µL H_2_O, and 60 µL Trolox + 240 µL H_2_O. After that Cu^2+^ working solution was prepared by diluting 1 part of Cu^2+^ Reagent with 49 parts of Assay Diluent 50 µL of Cu^2+^ added to 2450 µL of diluent. Finally, we prepared the serum by taking 3 µL of the serum for each mice group, and adding 297 µL H_2_O, then the well plate was prepared by adding 100 µL of each standard in each well and 100 µL of each sample in each well and adding 100 µL of Cu^2+^ working solution in all wells, after that by using plate mixer they were mixed well and incubated for 90 min, and read at 570 nm using microplate reader^23,29^.

### Porcine pancreatic lipase inhibition assay

Stock solutions of 500 µg/ml were made from each compound in 10% DMSO. From these, a dilution series of five concentrations of 50, 100, 200, 300, and 400 μg/ml were made. A 1 mg/ml stock solution of porcine pancreatic lipase in Tris–HCl buffer was prepared freshly just before use. The substrate, p-nitrophenyl butyrate (PNPB) was prepared by dissolving 20.9 mg in 2 ml acetonitrile. For each working solution, 0.1 ml porcine pancreatic lipase was mixed with 0.2 ml of compound solution from each member of the dilution series. Tris–HCl was added to make the final volume of the working solutions 1 ml, and they were incubated at 37 °C for 15 min. After incubation, 0.1 ml p-nitrophenyl butyrate solution was added to each test tube. The mixture was then incubated for a further 30 min at 37 °C. Pancreatic lipase activity was determined by measuring the hydrolysis of PNPB into p-nitrophenolate at 410 nm, using a UV spectrophotometer. The same procedure was repeated using Orlistat as a standard reference compound. Percentage lipase inhibition by compound dilution was calculated with the following equation:

$$ {\text{Lipase}}\;{\text{inhibition}}\;\% = \left( {{\text{A}}_{{\text{B}}} - {\text{A}}_{{{\text{ts}}}} } \right)/{\text{A}}_{{\text{B}}} \times 100\% $$

where, A_B_ is the recorded absorbance of the blank solution, and A_ts_ is the recorded absorbance of the tested sample solution^31^.

### α‑Amylase inhibitory assay

A 5 mg of each fraction was dissolved in a few milliliters of 10% DMSO, and then further dissolved up to 100 ml in 0.02 M Na_2_HPO_4_/NaH_2_PO_4_, 0.006 M NaCl, pH 6.9 to give finally stock solutions with concentrations of 1000 μg/ml. From these, the following dilutions were prepared of 10, 50, 70, 100, and 500 μg/ml, using 10% DMSO as the diluent. A 0.2 ml volume of 2 units/ml porcine pancreatic α-amylase was mixed with 0.2 ml compounds dilution and was incubated for 10 min at 30 °C. After incubation, 0.2 ml of a freshly prepared 1% starch solution in water was added, and the tubes were then incubated for at least three more minutes. At this point, the reaction was stopped by the addition of 0.2 ml 3,5-dinitro salicylic acid (DNSA) color reagent and was diluted with 5 ml of distilled water, before being heated at 90 °C for 10 min in a water bath. The mixture was then cooled to room temperature, and the absorbance was measured at 540 nm. The blank control was prepared using the same quantities described above, but replacing the compound solution with 0.2 ml buffer. Acarbose was used as a standard reference following the procedure described above. α-amylase inhibitory activity was calculated using the following equation:

$$ \% \;{\text{of}}\;{\upalpha } - {\text{amylase}}\;{\text{inhibition}} = \left( {{\text{A}}_{{\text{B}}} - {\text{A}}_{{\text{T}}} } \right)/{\text{A}}_{{\text{B}}} \times 100\% , $$

where, A_B_: is the absorbance of the blank sample, and A_T_ is the absorbance of the test sample^32^.

### Statistical analysis

Multiple mean values were compared using analysis of variance (ANOVA) with GraphPad Prism. Values presented are mean ± SD. ANOVA using *t*-tests was applied to compare the mean of each group with that of the control group. A *P* < 0.05 was considered to be statistically significant.

### Ethics approval and consent to participate

The study was carried out in accordance with the Helsinki Declaration. We declare that all authors approve the submission of the manuscript to this journal. The authors own the copyright for the entire manuscript, including all artwork and tables. The Institutional Review Board approved the study at the Faculty of Medicine and Health Sciences of An-Najah National University (Approve number: pharm. May 2012/15). As we as the study is reported in accordance with ARRIVE guidelines.

## Data Availability

The datasets used and/or analyzed during the current study are available from the corresponding author on reasonable request.

## Abbreviations

DMAP: Dimethylaminopyridine
DMF: Dimethylformamide
DMSO: Dimethyl sulfoxide
EDC: 1-Ethyl-3-(3-dimethylaminopropyl) carbodiimide
IC_50_: 50% Inhibition concentration
DNSA: 3,5-Dinitro salicylic acid

## References

[1] Vincent HK, Innes KE, Vincent KR (2007). Oxidative stress and potential interventions to reduce oxidative stress in overweight and obesity. Diabetes Obes. Metab..

[2] Ardeshirlarijani E, Tabatabaei-Malazy O, Mohseni S, Qorbani M, Larijani B, Jalili RB (2019). Effect of probiotics supplementation on glucose and oxidative stress in type 2 diabetes mellitus: A meta-analysis of randomized trials. DARU J. Pharm. Sci..

[3] Birben E, Sahiner UM, Sackesen C, Erzurum S, Kalayci O (2012). Oxidative stress and antioxidant defense. World Allergy Organ. J..

[4] Rana M, Katbamna R, Padhya A, Dudhrejiya A, Jivani N, Sheth N (2010). In vitro antioxidant and free radical scavenging studies of alcoholic extract of *Medicago sativa* L. Romanian J. Biol. Plant Biol..

[5] Poli G, Schaur JR (2000). 4-Hydroxynonenal in the pathomechanisms of oxidative stress. IUBMB Life.

[6] Rahman MM, Islam MB, Biswas M, Alam AK (2015). In vitro antioxidant and free radical scavenging activity of different parts of *Tabebuia pallida* growing in Bangladesh. BMC. Res. Notes.

[7] Kinnula VL, Crapo JD (2003). Superoxide dismutases in the lung and human lung diseases. Am. J. Respir. Crit. Care Med..

[8] Serban MC, Sahebkar A, Zanchetti A, Mikhailidis DP, Howard G, Antal D, Andrica F, Ahmed A, Aronow WS, Muntner P (2016). Effects of quercetin on blood pressure: A systematic review and meta-analysis of randomized controlled trials. J. Am. Heart Assoc..

[9] Hawash M, Eid AM, Jaradat N, Abualhasan M, Amer J, Zaid AN, Draghmeh S, Daraghmeh D, Daraghmeh H, Shtayeh T (2020). Synthesis and biological evaluation of benzodioxole derivatives as potential anticancer and antioxidant agents. Heterocycl. Commun..

[10] Eid AM, Hawash M, Amer J, Jarrar A, Qadri S, Alnimer I, Sharaf A, Zalmoot R, Hammoudie O, Hameedi S (2021). Synthesis and biological evaluation of novel isoxazole-amide analogues as anticancer and antioxidant agents. BioMed Res. Int..

[11] Hawash MM, Baytas SN (2018). Antiproliferative activities of some biologically important scaffolds. FABAD J. Pharm. Sci..

[12] Hawash M, Kahraman DC, Ergun SG, Cetin-Atalay R, Baytas SN (2021). Synthesis of novel indole-isoxazole hybrids and evaluation of their cytotoxic activities on hepatocellular carcinoma cell lines. BMC Chem..

[13] Hawash M, Jaradat N, Abualhasan M, Amer J, Levent S, Issa S, Ibrahim S, Ayaseh A, Shtayeh T, Mousa A (2021). Synthesis, chemo-informatics, and anticancer evaluation of fluorophenyl-isoxazole derivatives. Open Chem..

[14] Biava M, Battilocchio C, Poce G, Alfonso S, Consalvi S, Di Capua A, Calderone V, Martelli A, Testai L, Sautebin L (2014). Enhancing the pharmacodynamic profile of a class of selective COX-2 inhibiting nitric oxide donors. Bioorganic Med. Chem. J..

[15] Jeličić M-L, Kovačić J, Cvetnić M, Mornar A, Amidžić Klarić D (2022). Antioxidant activity of pharmaceuticals: Predictive QSAR modeling for potential therapeutic strategy. Pharmaceuticals.

[16] Winterbourn CC (1995). Toxicity of iron and hydrogen peroxide: The fenton reaction. Toxicol. Lett..

[17] Eid AM, Hawash M, Amer J, Jarrar A, Qadri S, Alnimer I, Sharaf A, Zalmoot R, Hammoudie O, Hameedi S (2021). Synthesis and biological evaluation of novel isoxazole-amide analogues as anticancer and antioxidant agents. Biomed Res. Int..

[18] Hawash M, Jaradat N, Bawwab N, Salem K, Arafat H, Hajyousef Y, Shtayeh T, Sobuh S (2021). Design, synthesis, and biological evaluation of phenyl-isoxazole-carboxamide derivatives as anticancer agents. Heterocycl. Commun..

[19] Hawash M, Jaradat N, Elaraj J, Hamdan A, Lebdeh SA, Halawa T (2019). Evaluation of the hypoglycemic effect of seven wild folkloric edible plants from Palestine:(Antidiabetic effect of seven plants from Palestine). J. Complement. Integr. Med..

[20] Hill R (1970). The chemistry of life: Eight lectures on the history of biochemistry.

[21] Stenesh J (1998). Biochemistry 2.

[22] Hussain S, Taha M, Rahim F, Hayat S, Zaman K, Iqbal N, Selvaraj M, Sajid M, Bangesh MA, Khan F (2021). Synthesis of benzimidazole derivatives as potent inhibitors for α-amylase and their molecular docking study in management of type-II diabetes. J. Mol. Struct..

[23] Somparn N, Saenthaweeuk S, Naowaboot J, Thaeomor A, Kukongviriyapan V (2018). Effect of lemongrass water extract supplementation on atherogenic index and antioxidant status in rats. Acta Pharm..

[24] Hawash M, Kahraman DC, Olgac A, Ergun SG, Hamel E, Cetin-Atalay R, Baytas SN (2022). Design and synthesis of novel substituted indole-acrylamide derivatives and evaluation of their anti-cancer activity as potential tubulin-targeting agents. J. Mol. Struct..

[25] Cheung L, Cheung PC, Ooi VE (2003). Antioxidant activity and total phenolics of edible mushroom extracts. Food Chem.

[26] Sandouka S, Shekh-Ahmad T (2021). Induction of the Nrf2 pathway by sulforaphane is neuroprotective in a rat temporal lobe epilepsy model. Antioxidants.

[27] Rahhal, B., Hattab, S., Jaradat, N., Basha, W., Al Zabadi, H., Zyoud, A., Taha, I., Najajreh, I., Ghanim, M. Phytochemical investigation and diuretic activity of the Palestinian rataegus aronia in mice using an aqueous extract. *Pal. Med. Pharm. J.* **7**(2), None-None (2021).

[28] Nabavi SM, Nabavi SF, Eslami S, Moghaddam AH (2012). In vivo protective effects of quercetin against sodium fluoride-induced oxidative stress in the hepatic tissue. Food Chem..

[29] Ruigrok S, Yim K, Emmerzaal T, Geenen B, Stöberl N, den Blaauwen J, Abbink M, Kiliaan A, van Schothorst E, Kozicz T (2021). Effects of early-life stress on peripheral and central mitochondria in male mice across ages. Psychoneuroendocrinology.

[30] Ko MJ, Mulia GE, Van Rijn RM (2019). Commonly used anesthesia/euthanasia methods for brain collection differentially impact MAPK activity in male and female C57BL/6 mice. Front. Cell. Neurosci..

[31] Drent M, Larsson I, William-Olsson T, Quaade F, Czubayko F, Von Bergmann K, Strobel W, Sjöström L, Van der Veen E (1995). Orlistat (Ro 18–0647), a lipase inhibitor, in the treatment of human obesity: A multiple dose study. Int. J. Obes. Relat. Metab. Disord..

[32] Sudha P, Zinjarde SS, Bhargava SY, Kumar AR (2011). Potent α-amylase inhibitory activity of Indian ayurvedic medicinal plants. BMC Complement Altern. Med..

